# Clinical management of CADASIL in Europe: bridging evidence gaps and harmonizing care – a consensus review

**DOI:** 10.1016/j.cccb.2026.100571

**Published:** 2026-11-04

**Authors:** Nicola Rifino, Julie Rutten, Gido Gravesteijn, Anna Bersano, Stephanie Guey, Dominique Hervè, Ana María Domínguez Mayoral, Anne Hege Nymoen Aamodt, Hugues Chabriat, Saskia Lesnik Oberstein

**Affiliations:** a Department of Clinical Genetics, Leiden University Medical Center, Leiden, the Netherlands; b Cerebrovascular Unit, Fondazione IRCCS Istituto Neurologico "Carlo Besta", Milan, Italy; c Translational Neurovascular Centre and CERVCO, VASCERN, APHP, Hôpital Lariboisière, Université Paris Cité, FHU Neuro-Vasc, Paris, France; d Department of Neurology, Virgen Macarena University Hospital, Seville, Spain; e Department of Neurology, Oslo University Hospital, Oslo, Norway; f Department of Neuromedicine and Movement Science, Norwegian University of Science and Technology (NTNU), Trondheim, Norway; g Institute of Population Health, Department of Primary Care and Mental Health, University of Liverpool, Liverpool, UK

**Keywords:** CADASIL, NOTCH3, Leukoencephalopathy, Small vessel disease, Stroke, Migraine, Neurogenetics

## Abstract

Cerebral autosomal dominant arteriopathy with subcortical infarcts and leukoencephalopathy (CADASIL) is the most common monogenic cause of cerebral small vessel disease, yet its clinical management remains poorly harmonized across Europe. This consensus review, developed on behalf of the NEUROVASC Working Group, examines current diagnostic and management practices for CADASIL in Europe and identifies priority areas for evidence generation and care harmonization. We describe the geographic distribution of pathogenic *NOTCH3* variants across European populations, the organization of referral pathways and specialized care networks, and current approaches to neuroimaging staging, genetic testing, and long-term follow-up. Management of major clinical manifestations is reviewed, including antithrombotic therapy and thrombolysis in acute stroke, cardiovascular risk factor control, migraine treatment, and cognitive and neuropsychiatric symptoms, areas in which practice is largely extrapolated from sporadic small vessel disease in the absence of disease-specific trials or guidelines. Closing these gaps will require multicenter registries and harmonized care pathways, validated outcome measures such as standardized MRI tools and disease severity staging, greater integration of patient-reported outcomes, and coordinated international collaboration. These efforts will help leverage the statistical power, genetic diversity, and clinical insights needed to advance-based, precision medicine approaches for patients with CADASIL.

## Highlights

• Care for CADASIL patients remains fragmented and poorly harmonized across Europe.
• Cognitive and psychiatric symptoms in CADASIL lack effective treatment options.
• Antithrombotic and thrombolytic therapy in CADASIL lack disease-specific evidence.
• No standardized European guidelines exist for CADASIL surveillance.
• Expanding access, standardizing care, and patient engagement are key priorities.

## 1 Introduction

Cerebral small vessel disease (cSVD) is a major contributor to stroke, long-term disability, and cognitive decline worldwide [1]. Among monogenic forms of cSVD, cerebral autosomal dominant arteriopathy with subcortical infarcts and leukoencephalopathy (CADASIL), caused by cysteine-altering variants in the *NOTCH3* gene, is the most common. Although the minimum prevalence of clinically recognized CADASIL is estimated at approximately 4 cases per 100,000 individuals [[2], [3], [4], [5]], population-based studies indicate that pathogenic *NOTCH3* variants occur in approximately 1:300 individuals worldwide [3], suggesting a broader disease spectrum and considerable underdiagnosis in routine clinical practice [[6], [7], [8]]. Clinically, classical CADASIL is characterized by recurrent subcortical ischemic events, progressive cognitive decline, and ultimately vascular dementia. Migraine with aura and neuropsychiatric symptoms, particularly mood disorders, are also frequent. Neuroimaging abnormalities, notably white matter hyperintensities, often precede the clinical symptoms by several years to decades [9].

Despite advances in genetic and radiological characterization, clinical management of CADASIL remains poorly harmonized across Europe for several reasons. One major challange is the highly variable and difficult to predict disease trajectory: although the position of the pathogenic *NOTCH3* variant within the 34 epidermal growth factor–like repeat (EGFr) domains has emerged as an important prognostic factor, individual outcome prediction remains imprecise [8] as other modifiers also play a role such as conventional cardiovascular risk factor burden and also still unknown modifiers [7,10].

Currently there are no disease modifying treatments for CADASIL and pharmacological treatment strategies are largely extrapolated from sporadic cSVD rather than grounded in disease-specific evidence [11,12], resulting in scientific statements [13] and consensus recommendations [14] rather than formal guidelines for CADASIL based on clinical trial-based evidence.

Beyond these knowledge gaps, important disparities persist between European countries in disease awareness, access to specialized and multidisciplinary care, and availability of research infrastructure. Together, these factors sustain a landscape of fragmented, expert-dependent clinical practice. The European Academy of Neurology (EAN) has highlighted the need for a strategic, patient-centered research agenda to address these challenges in rare neurological diseases, including CADASIL [15,16].

This review provides an overview of current clinical management practices for patients with CADASIL across Europe, identifies key gaps in evidence and care delivery and outlines priorities for developing more harmonized, patient-centered, and evidence-based approaches.

## 2 Spectrum of *NOTCH3* variants in the European population

Understanding the geographic distribution of pathogenic *NOTCH3* variants is clinically relevant for European physicians. Since variant position within the 34 EGFr-domains is the strongest determinant of disease severity [6,8], the local prevalence of high-, moderate-, and low-risk variants directly shapes the expected disease burden across different European centres [8]. Depending on the predominance of specific *NOTCH3* variants within and between countries, disease severity may vary substantially according to these genetic-geographical differences. The most commonly reported variants in European CADASIL populations include the high-risk variants p.Arg90Cys, p.Arg141Cys, p.Arg169Cys, p.Arg182Cys, p.Arg207Cys and p.Arg1006Cys, though their relative frequencies differ considerably by region [17]. Northern European populations, including the UK [10,18], France [9,19], and the Netherlands [8,20], have many *NOTCH3* variants in common, with a predominance of high-risk variants. The most striking European example of a *NOTCH3* variant with a founder effect has been reported in Finland, where a single variant accounts for nearly 70% of cases [21]. Region-specific clustering has similarly been hypothesised in central and northeastern Italy [22,23]. By contrast, Portugal, the Netherlands, and Slovakia display exceptional mutational heterogeneity, with numerous distinct variants, suggesting multiple independent mutational events [24,25].

## 3 Organization of care and referral pathways in Europe

The organization of care for CADASIL patients in Europe is characterized by heterogeneity in standards of care across countries. In many European countries, CADASIL patients are managed through a combination of local neurology services and academic expert centers, often embedded in services with expertise in cerebral SVD, rare neurological disorders, or rare genetic disorders. Referral pathways typically involve initial evaluation by general neurologists or stroke physicians, followed by referral to specialized centers for genetic confirmation, advanced neuroimaging, and multidisciplinary management [26].

A European Stroke Organization (ESO) scientific seminar in 2025 highlighted the need for dedicated small vessel disease clinics that integrate vascular risk factor management, cognitive assessment, neuropsychiatric care, and patient education, a model particularly applicable to CADASIL and other monogenic SVDs [26]. Several NEUROVASC partner centres already operate dedicated CADASIL or monogenic SVD clinics embedding these components, representing existing hubs of expertise that could serve as models for broader implementation across Europe [27].

The European Reference Network facilitates a European infrastructure for coordinating CADASIL care across Europe. Since 2022, CADASIL has been included within the NEUROVASC working group, currently comprising full members from Italy, the Netherlands, France, and Germany, with developing partners in Spain, Norway, and Czech Republic. The network facilitates virtual case discussions through the Clinical Patient Management System (CPMS), a secure web-based platform enabling clinicians to share diagnostically challenging cases, share neuroimaging, and obtain expert opinions from multiple centers across Europe [28].

To further harmonize the diagnostic process, VASCERN experts have developed and published a CADASIL patient pathway (Fig. 1) [29], providing a standardized referral and diagnostic framework applicable across different European healthcare systems.

**Fig. 1:**
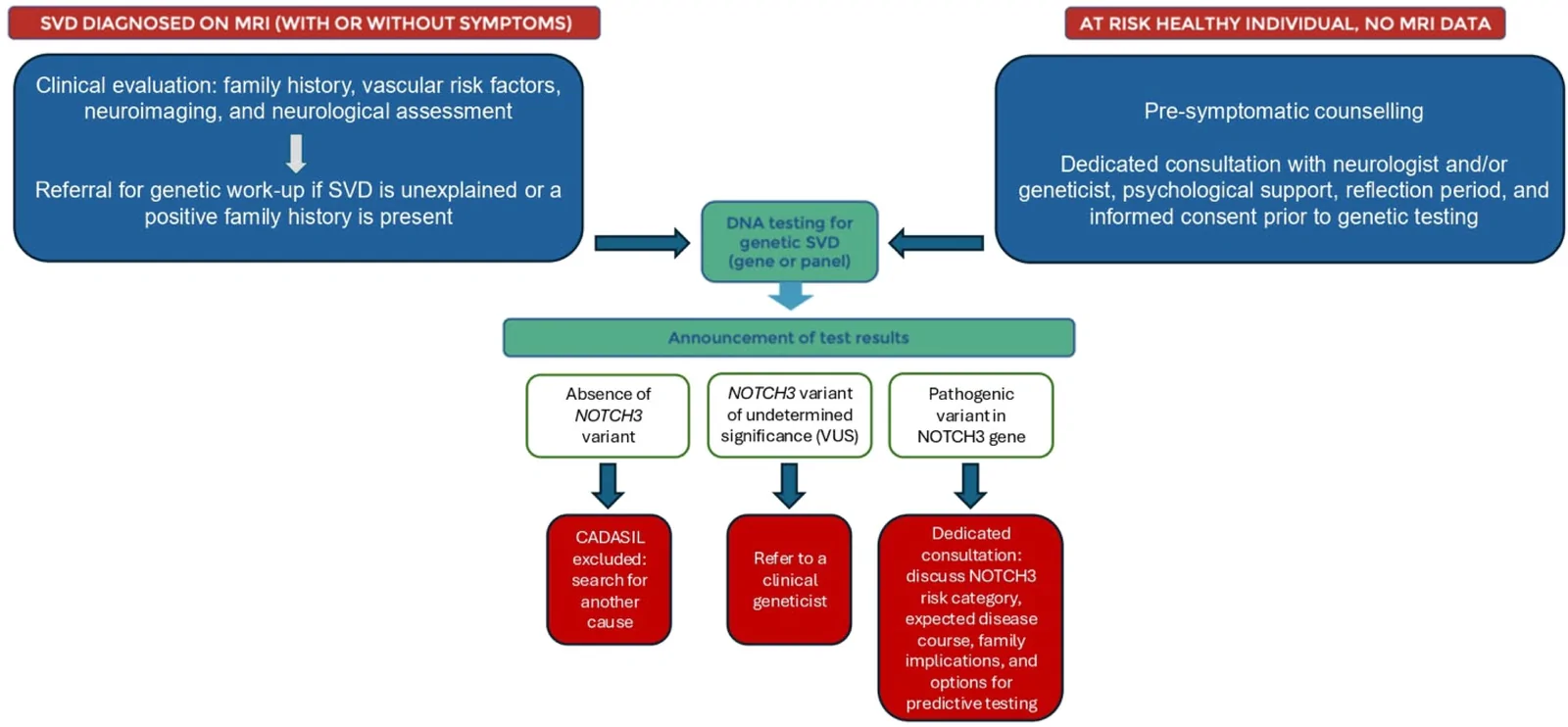
Simplified CADASIL clinical pathway. Adapted and simplified from the VASCERN CADASIL Patient Pathway (2024) [30], this figure outlines the key steps in the diagnostic workup and management of patients with suspected CADASIL.

## 4 Diagnostic work-up and follow-up in clinical practice

The diagnostic work-up of CADASIL is ideally conducted within a multidisciplinary centre specialised in rare cerebrovascular diseases. CADASIL should be considered in the differential diagnosis of patients presenting with a relatively high load of white matter hyperintensities (WMH) on T2-weighted MR images, with or without other imaging markers of SVD. Suggestive family history and a characteristic clinical presentation should also arise the suspicion of CADASIL. However, given the broad phenotypic spectrum of NOTCH3-SVD, disease expression can be highly variable even within families, and diagnostic delays remain common, particularly in patients with atypical presentations or late-onset disease.

### 4.1 Neuroimaging

Brain MRI is a prerequisite for determining the NOTCH3-SVD stage, for which Fazekas score and lacune count are needed. This staging system was developed and validated in an international cohort of patients with CADASIL, and ranges from premanifest with normal brain MRI (stage 0) to severe disability with high MRI lesion burden (stage 4A) [6]. (Fig. 2)

**Fig. 2:**
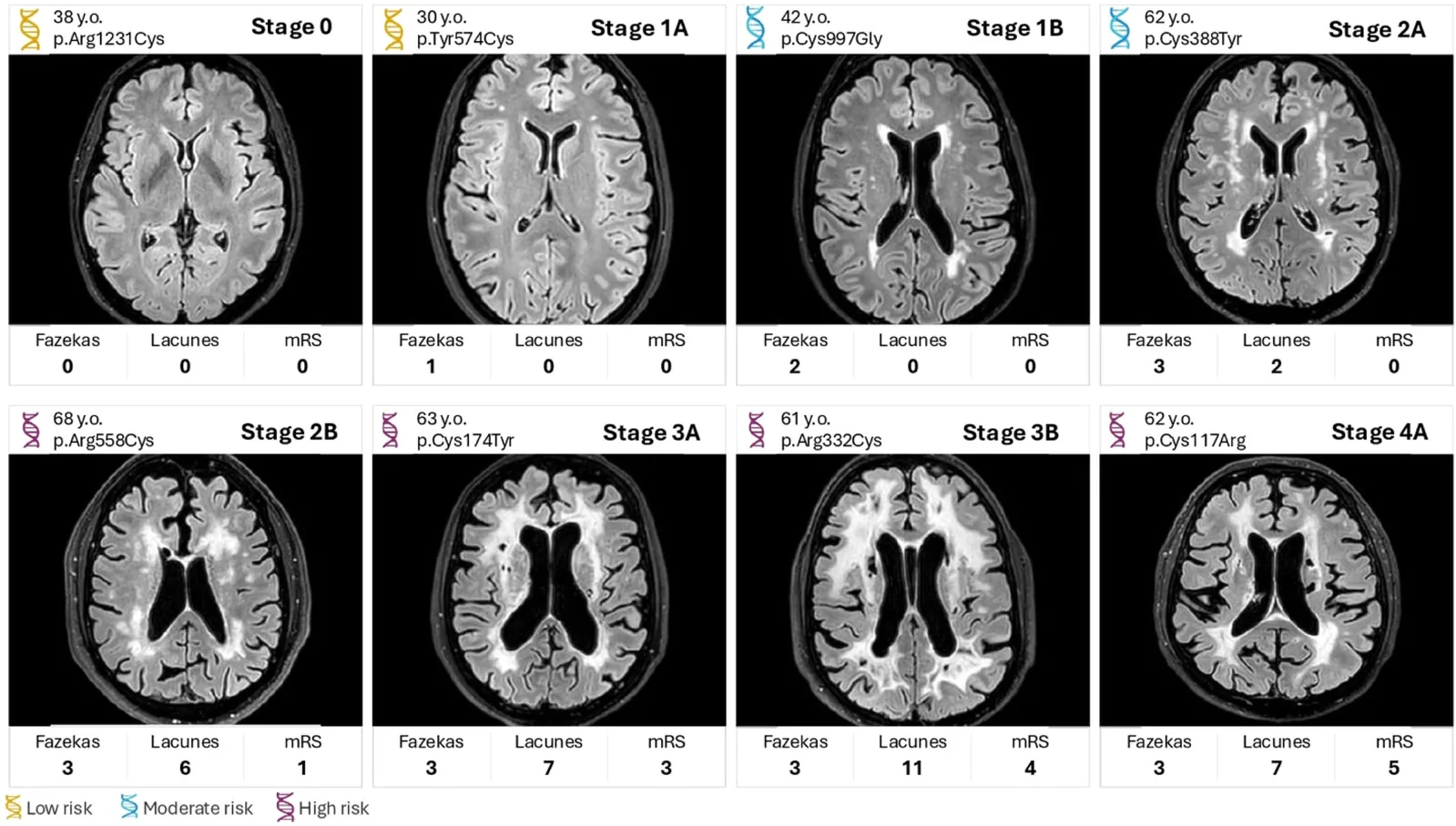
**Genetic, clinical and neuroimaging profiles across NOTCH3-SVD disease stages.** Axial FLAIR images illustrating white matter hyperintensity burden, lacune load, and functional disability (mRS) across Stages 0–4A, alongside the causative NOTCH3 variant and its EGFr risk category (DNA strand icons: gold = low-risk, teal = moderate-risk, purple = high-risk).

MR-imaging should preferably include T1-weighted, T2-weighted, and FLAIR sequences, which allow for the detection of white matter hyperintensities (WMH), lacunes, and brain atrophy, while susceptibility-weighted imaging (SWI) or T2-weighted gradient-recalled echo sequences are used to assess the presence of cerebral microbleeds (CMB) [13]. A comprehensive MRI evaluation should ideally also include diffusion-weighted imaging for identifying recent infarcts.

Beyond symmetrical high WMH lesion burden, the topographical distribution of lesions can be informative as patients with CADASIL relatively frequently show involvement of the anterior temporal lobes and external capsules, although in many patients WMH in these locations are absent. CADASIL can therefore be difficult to distinguish from sporadic small vessel disease especially in older patients (Fig. 3) [13].

**Fig. 3:**
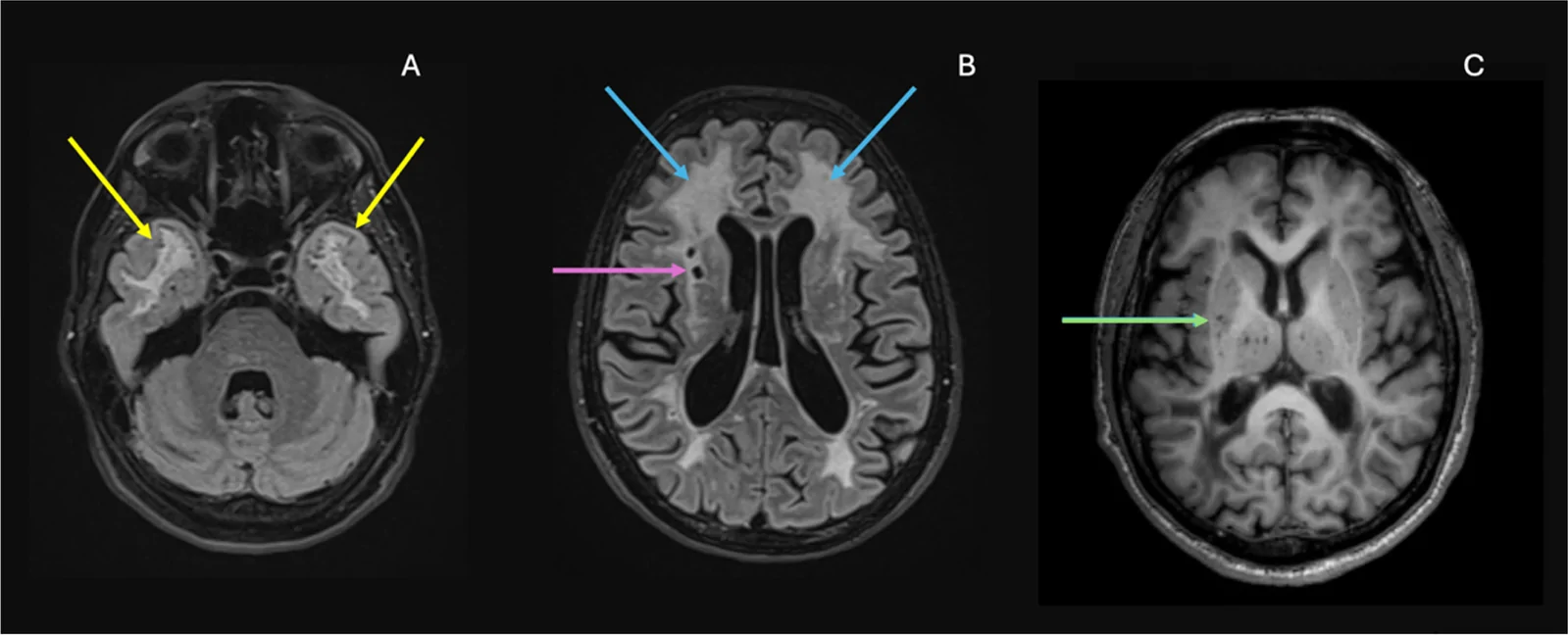
MRI in CADASIL **(A)** Bilateral hyperintense lesions involving the anterior temporal lobes (yellow arrows). **(B)** Confluent white matter hyperintensities in the frontal periventricular and subcortical regions (blue arrows), extending toward the external capsule, with associated subcortical lacunes (purple arrow). **(C)** T1-weighted axial image showing dilated perivascular spaces in the deep gray matter (green arrow).

To address the need for cross-cohort comparisons in CADASIL, the CADA-MRIT (CADASIL MRI Inventory Tool) was recently developed and validated across French, German, and Taiwanese cohorts [31]. Such standardized tools may facilitate harmonized imaging recommendations across centers and enable more robust longitudinal assessment and multicenter research collaboration.

### 4.2 Genetic testing strategies

Genetic confirmation through identification of a pathogenic *NOTCH3* variant remains the gold standard for the diagnosis of CADASIL. Across Europe, genetic testing strategies differ substantially, reflecting variations in laboratory infrastructure, and reimbursement policies [32]. In some countries, targeted sequencing of *NOTCH3* exons encoding EGFr domains is still routinely performed, whereas other centers increasingly rely on gene panels for monogenic small vessel diseases or for broader neurological disorders.

Regardless of the sequencing approach, complete coverage of all exons encoding the 34 EGFr domains is recommended to avoid missing pathogenic variants. Whole-exome sequencing may be used in selected cases with atypical phenotypes or negative targeted testing [33]. The above testing strategy applies to symptomatic individuals presenting with a clinical and neuroimaging picture suggestive of CADASIL. For at-risk family members of a known index case, directed sequencing targeting the familial variant is sufficient for predictive DNA testing.

Regardless of the method used, interpretation of genetic results poses additional challenges, particularly in the case of variants of uncertain significance such as most non-cysteinealtering variants [[34], [35], [36], [37]]. Skin biopsy showing vessel wall granular osmiophilic material (GOM) on electron microscopy and positive NOTCH3 immunostaining provides supportive diagnostic evidence.

### 4.3 Long-term monitoring and follow-up

Long-term follow-up of patients with CADASIL aims to monitor disease progression, manage complications, and provide anticipatory guidance. No standard European surveillance guidelines for CADASIL exist, and most follow-up protocols are based on expert opinion rather than prospective data.

#### 4.3.1 Presymptomatic patients

The management of presymptomatic *NOTCH3*-positive individuals presents one of the most debated aspects of CADASIL care. In current practice, surveillance strategies vary markedly, reflecting the absence of disease-modifying therapies and limited evidence for the benefits of early monitoring and primary stroke prevention. Some centers offer regular neurological evaluations and MRI surveillance, whereas others adopt a more conservative approach, focusing on vascular risk factor control and patient education.

Psychological support and counseling are particularly important in this population, given the emotional burden associated with a presymptomatic genetic diagnosis and uncertainty regarding disease onset and progression. Integrating psychologists into multidisciplinary teams at CADASIL reference centers could help standardize access to psychological care.

#### 4.3.2 Symptomatic patients

Symptomatic patients are generally followed within specialized neurology or cerebrovascular clinics, often at referral centers with expertise in CADASIL. Follow-up typically focuses on secondary stroke prevention, management of cognitive and neuropsychiatric symptoms, migraine control, and functional status assessment, as well as psychological support, if available. The frequency of clinical evaluations depends on NOTCH3-SVD stage, the severity and type of symptoms and the needs of patients and their caregivers, ranging from low-frequency follow-up in stable patients to more frequent evaluations in those with rapid progression or significant disability.

## 5 Management of clinical manifestations

### 5.1 Management of stroke

#### 5.1.1 Role of antiplatelet and anticoagulation

There are no randomized controlled trials or disease-specific guidelines for antithrombotic therapy in CADASIL. Current practice is largely extrapolated from sporadic small vessel disease, [13,14] but it varies considerably across centres. Antiplatelet agents are commonly prescribed after ischemic stroke or transient ischemic attack in CADASIL, although this is not evidence-based [14,38]. In Japan, for instance, nearly 74% of CADASIL patients receive antiplatelet therapy [39]. Dual antiplatelet therapy is usually avoided due to the hypothesized increased risk of intracerebral hemorrhage, particularly in patients with cerebral microbleeds [37,39].

Anticoagulation is not routinely recommended in CADASIL and is typically restricted to patients with clear alternative indications, such as atrial fibrillation, after careful individual risk–benefit assessment [13,14]. In selected patients with high CHA_2_DS_2_-VASc scores and high microbleed count, left atrial appendage closure may be considered as an alternative to long-term anticoagulation [13].

#### 5.1.2 Role of thrombolytic therapies

Intravenous thrombolysis with recombinant tissue plasminogen activator represents standard care for eligible patients with acute ischemic stroke in the general population [40]. However, its application in CADASIL remains controversial due to the disease’s unique pathophysiology and theoretical hemorrhagic risks [41]. Published experience with thrombolysis in CADASIL remains extremely limited, consisting primarily of case reports and small case series that preclude robust conclusions about safety or efficacy [[41], [42], [43]]. Significant questions about thrombolytic efficacy in CADASIL-related strokes have been raised, as CADASIL-related strokes are almost exclusively a consequence of intrinsic small vessel arteriopathy rather than large vessel occlusion or cardioembolism [44]. Furthermore, in the general stroke population, the presence of >10 cerebral microbleeds increases post-thrombolysys intracerebral hemorrhage risk approximately 12-fold compared to patients with ≤10 microbleeds, [45] while the presence of WMH independentely doubles the risk of post-thrombolysis hemorrhage [46]. This raises particular concern in the CADASIL population in which both imaging findings are prevalent. Consequently, this supports a cautious and individualized approach to decision-making regarding thrombolysis in CADASIL. However, in cases of large-vessel occlusion related to a coexisting cardioembolic or atherothrombotic disease, decisions regarding reperfusion therapy should be made on an individual basis, taking into account the burden of underlying small vessel disease markers, especially cerebral microbleeds, as well as any prior intracerebral hemorrhage. In this setting, mechanical thrombectomy, with or without intravenous thrombolysis, may reasonably be considered on an individual basis in a CADASIL patient with large vessel stroke, given its established efficacy in large vessel occlusion stroke in the general population.

#### 5.1.3 Cerebrovascular risk factor management

The management of cerebrovascular risk factors represents a cornerstone of clinical care in CADASIL, as it has been shown that a high cardiovascular risk factor burden is associated with more severe disease [7,47,48].

Hypertension and smoking have been found to be strongly associated with worse outcomes in CADASIL, including incident stroke, intracerebral hemorrhage, dementia, and cerebral microhemorrhages [7,47,[49], [50], [51]]. Strict blood pressure monitoring is therefore advised to all patients with CADASIL, and the importance of smoking cessation should be strongly emphasized[13]. In contrast, the role of lipid management in CADASIL remains uncertain. No clear association between hyperlipidemia and clinical outcomes has been established, and a small study of short-term atorvastatin treatment showed no significant improvement in hemodynamic parameters in CADASIL patients [52]. To date, there is no compelling evidence for or against the prescription of statins for secondary prevention following a stroke. However, we do not yet know whether this treatment offers any real benefit to patients, nor what LDL cholesterol targets should be recommended [14].

#### 5.1.4 Preventive therapeutic options

When considering preventive therapies, it remains unclear whether patients with a confirmed diagnosis of CADASIL who have never had a stroke or transient ischemic attack should routinely receive antiplatelet therapy, statins, and/or antihypertensive drugs.

Routine prescription of antiplatelet for primary prevention is not recommended, given the lack of proven efficacy and the potential bleeding risk associated with small vessel disease [29,53]. Similarly, there is no evidence supporting the routine use of statins in this setting. Statins and antihypertensive drugs should instead be prescribed according to standard indications, such as dyslipidemia or hypertension, in line with general cardiovascular prevention guidelines [29,54]. Counseling on smoking cessation is beneficial to reduce the risk of incident stroke [53].

However, individual clinical and neuroimaging circumstances may justify a different management strategy. In selected patients with a higher NOTCH3-SVD stage [6], preventive treatment may be considered on a case-by-case basis, after weighing the potential benefit against the bleeding risk, including microbleed burden and prior intracerebral hemorrhage (ICH), and after shared decision-making with the patient.

### 5.2 Management of migraine

Migraine with aura is a common and often early manifestation of CADASIL, occurring in up to 50% of patients, a prevalence five times higher than in the general population [9,55,56]. Based on limited evidence, triptans and ergot derivatives are considered to be safe and effective [14]. Nonetheless, conventional analgesics and nonsteroidal anti-inflammatory drugs are preferred over vasoconstrictors by some, reflecting ongoing debate about theoretical risks [9]. NEUROVASC recommendations are to use triptans as a second or third-choice option, despite the absence of contraindications [29]. In this context, lasmiditan, a selective 5-HT1F receptor agonist, has been proposed as a theoretically safer alternative for acute migraine treatment in patients with vascular comorbidities. However, no data exist in CADASIL patients, and there is currently insufficient evidence to recommend it over other treatments.

Prophylactic options for CADASIL remain poorly supported by evidence. A meta-analysis found most common choices equivocal in efficacy, with propranolol potentially deleterious; acetazolamide and sodium valproate were considered physiologically plausible [57]. Acetazolamide showed some benefit in a small case series and demonstrated improved cerebral hemodynamics after 24 weeks [58]. Amitriptyline is used empirically but should be avoided with caution in cognitively impaired patients given its anticholinergic profile. Other suggested options include sodium valproate, lamotrigine, topiramate, verapamil, and flunarizine [57].

The advent of novel migraine therapies has introduced specific concerns regarding cerebrovascular safety in CADASIL. No robust data exist on the benefit of CGRP (Calcitonin gene-related peptide) modulators in CADASIL and their use in CADASIL remains controversial due to theoretical safety concerns. CGRP serves as a vasodilatory safeguard during cerebral and cardiac ischemia, and blocking this system could potentially worsen ischemic events in patients with underlying small vessel disease [59]. A 2020 expert opinion cautioned against treating CADASIL patients with CGRP-blocking drugs until relevant safety data and long-term follow-up results become available [59]. However, recent case reports challenge this cautious stance, showing significant reduction in attack frequency and pain intensity, with improvement in quality of life and no adverse effects in two cases [60]. Non-pharmacological and adjunctive options, including onabotulinumtoxinA for chronic migraine, peripheral nerve blocks, acupuncture, and wearable neuromodulation devices, have not been specifically evaluated in CADASIL-related migraine; however, given their generally favorable safety profile, they may represent reasonable therapeutic options to consider on an individual basis, extrapolating from their use in the general migraine population [61].

### 5.3 Management of cognitive decline and psychiatric manifestations

Cognitive impairment is typically characterized by a progressive decline in executive functions, including attention, processing speed, planning, and mental flexibility, reflecting the predominant involvement of subcortical-frontal networks [62]. Memory complaints are also common, although they are often secondary to deficits in attention and retrieval [62]. Alongside cognitive decline, a wide range of psychiatric symptoms may emerge, most commonly including depression [9,13,62,63], along with anxiety symptoms and apathy, which is often associated with functional decline, and increased caregiver burden [62,64,65]. Management of depression generally follows conventional pharmacotherapy, such as selective serotonin reuptake inhibitors [13]. However, treatment response sometimes remains incomplete, and symptoms refractory [62,66,67]. Beyond mood disturbances, pharmacologic interventions for cognitive symptoms remain limited. A randomized controlled trial of donepezil in CADASIL showed at 90 days follow up no benefit on global cognition but did demonstrate improvement in some executive function subscores [68].

## 6 Other clinical manifestations of cadasil

Beyond the classical triad of migraine with aura, ischemic stroke, and progressive cognitive decline, CADASIL encompasses a spectrum of less common manifestations that carry specific diagnostic and management implications despite the absence of disease-specific evidence.

*Acute encephalopathy.* Acute confusional episodes with reduced arousal, should initially be approached as a medical and neurological emergency, with exclusion of infection/meningoencephalitis, metabolic or toxic causes, acute ischemic or hemorrhagic stroke, and nonconvulsive status epilepticus. In a large British national cohort of 340 symptomatic CADASIL patients, encephalopathy occurred in 10.3% and complete recovery within three months was observed in 96% of episodes, supporting a typically self-limited natural history [69]. No CADASIL-specific acute therapy has been established, and supportive management is generally appropriate.

*Seizures and epilepsy.* Epilepsy is uncommon in CADASIL. In a cohort of 446 patients, seizures were reported in 8.6% [19]. Management follows standard epilepsy practice, with antiseizure medication selected according to seizure type and attention to interactions and comorbid mood or cognitive symptoms.

*Intracerebral hemorrhage and hemorrhage-risk states.* Acute ICH prevalence in CADASIL varies considerably between European and Asian cohorts [70,71]. In predominantly European series, symptomatic ICH has generally been reported in the range of 0.5–2%, whereas East Asian cohortsreport substantially higher rates [70,71]. The reasons for this difference remain unclear. Genetic background, environmental factors, or a combination of both may contribute. ICH should be managed according to standard ICH protocols, as CADASIL-specific evidence is limited. With respect to secondary prevention, anticoagulant exposure is associated with a significantly higher risk of ICH, whereas this does not seem to be the case for antiplatelet exposure [71].

*Movement-disorder phenotypes.* Vascular parkinsonism and, less commonly, atypical parkinsonian or chorea/dystonia phenotypes have been described, generally attributed to cumulative subcortical small vessel disease burden; response to levodopa is often limited in reported cases, so management typically emphasizes rehabilitation, gait and balance therapy, with cautious use of medication where appropriate [[72], [73], [74]].

## 7 Patient advocacy

In the absence of disease-modifying therapies, the management of CADASIL relies heavily on informed and proactive patient involvement. Recognizing this need, the VASCERN, through its NEUROVASC working group, has tried to translate expert consensus into practical, patient-oriented recommendations, aimed at supporting both clinicians and patients in everyday disease management. Practical “Do’s and Don’ts” for CADASIL have been published, providing accessible guidance on lifestyle modifications, and symptom management [29].

## 8 Conclusions

The clinical management of CADASIL remains largely supportive and is constrained by the absence of disease-specific therapies and limited high-quality evidence.

Future priorities should include the development of multicenter registries and harmonized care pathways to enable longitudinal follow-up, facilitate research, and support the identification of biomarkers for disease progression and treatment response. International collaboration is critical for conducting adequately powered clinical trials and advancing targeted research into disease mechanisms and potential therapies.

Looking ahead, closing these gaps will require not only advances in disease-modifying therapies and harmonized care pathways, but also a greater integration of the patient perspective. Patient-reported outcomes are increasingly recognized as essential endpoints in rare disease research, and their incorporation into future CADASIL clinical trials will be critical to capture the full impact of the disease on daily functioning and quality of life [75].

In summary, coordinated efforts to expand access, standardize care, and integrate patient engagement are essential for improving CADASIL patient care. International collaborations have resulted in important advances, including a validated MRI tool [31], improved genetic risk stratification [8], and a disease severity staging system [6], enabling uniform data collection across centers worldwide. By fostering coordinated international efforts, we can leverage the statistical power, genetic diversity, and clinical insights needed to advance precision medicine for patients with CADASIL.

## CRediT authorship contribution statement

**Nicola Rifino:** Writing – review & editing, Writing – original draft, Conceptualization. **Julie Rutten:** Writing – review & editing, Supervision, Conceptualization. **Gido Gravesteijn:** Writing – review & editing, Supervision. **Anna Bersano:** Writing – review & editing. **Stephanie Guey:** Writing – review & editing. **Dominique Hervè:** Writing – review & editing. **Ana María Domínguez Mayoral:** Writing – review & editing. **Anne Hege Nymoen Aamodt:** Writing – review & editing. **Hugues Chabriat:** Writing – review & editing. **Saskia Lesnik Oberstein:** Writing – review & editing, Validation, Supervision, Methodology, Conceptualization.

## Declaration of competing interest

The authors declare that they have no known competing financial interests or personal relationships that could have appeared to influence the work reported in this paper.
